# Identifying Patient–Ventilator Asynchrony on a Small Dataset Using Image-Based Transfer Learning

**DOI:** 10.3390/s21124149

**Published:** 2021-06-17

**Authors:** Qing Pan, Mengzhe Jia, Qijie Liu, Lingwei Zhang, Jie Pan, Fei Lu, Zhongheng Zhang, Luping Fang, Huiqing Ge

**Affiliations:** 1 College of Information Engineering, Zhejiang University of Technology, Liuhe Rd. 288, Hangzhou 310023, China; pqpq@zjut.edu.cn (Q.P.); 2111903025@zjut.edu.cn (M.J.); 2112003021@zjut.edu.cn (Q.L.); 2111803020@zjut.edu.cn (L.Z.); 2111903016@zjut.edu.cn (J.P.); luf@zjut.edu.cn (F.L.); 2 Department of Emergency Medicine, Sir Run Run Shaw Hospital, School of Medicine, Zhejiang University, Qingchun East Rd. 3, Hangzhou 310016, China; zh_zhang1984@zju.edu.cn; 3 Department of Respiratory Care, Regional Medical Center for National Institute of Respiratory Diseases, Sir Run Run Shaw Hospital, School of Medicine, Zhejiang University, Qingchun East Rd. 3, Hangzhou 310016, China

**Keywords:** mechanical ventilation, transfer learning, deep learning, patient–ventilator asynchrony, convolutional neural network

## Abstract

Mechanical ventilation is an essential life-support treatment for patients who cannot breathe independently. Patient–ventilator asynchrony (PVA) occurs when ventilatory support does not match the needs of the patient and is associated with a series of adverse clinical outcomes. Deep learning methods have shown a strong discriminative ability for PVA detection, but they require a large number of annotated data for model training, which hampers their application to this task. We developed a transfer learning architecture based on pretrained convolutional neural networks (CNN) and used it for PVA recognition based on small datasets. The one-dimensional signal was converted to a two-dimensional image, and features were extracted by the CNN using pretrained weights for classification. A partial dropping cross-validation technique was developed to evaluate model performance on small datasets. When using large datasets, the performance of the proposed method was similar to that of non-transfer learning methods. However, when the amount of data was reduced to 1%, the accuracy of transfer learning was approximately 90%, whereas the accuracy of the non-transfer learning was less than 80%. The findings suggest that the proposed transfer learning method can obtain satisfactory accuracies for PVA detection when using small datasets. Such a method can promote the application of deep learning to detect more types of PVA under various ventilation modes.

## 1. Introduction

Mechanical ventilation (MV) is one of the most important life-support treatments for patients who are unable to breathe on their own. It is used to assist patients during rest and recovery from their primary diseases [1]. Patient–ventilator asynchrony (PVA) occurs when the phases of the breath delivered by the ventilator do not match that of the patient’s respiratory center output or when there is a mismatch between the demands of the patient and the assistance offered by the ventilator [2,3]. This phenomenon is associated with a series of adverse clinical outcomes, such as the failed liberation of patients from ventilators [4,5], extended stays in intensive care units (ICU), increased likelihood of respiratory-muscle and diaphragmatic injuries, and increased consumption of sedatives and paralytics [6]. Studies have demonstrated that PVA can be detected by analyzing airway pressure, flow velocity, and tidal volume sequences [3,7]. Traditional PVA detection methods involve observing and evaluating the respiratory sequences at the bedside [8]. However, because many clinicians have insufficient recognition ability [9] and cannot remain at the bedside long-term, this detection method still has clinical limitations.

Some computerized algorithms have been developed for PVA detection over the past decade [10]. Early efforts include rule-based algorithms that computed features from the respiratory sequences and set thresholds to identify PVA cycles [1,11]. In recent years, machine learning has been used for the detection of PVA. By manually selecting valuable features and inputting them into machine learning models, such as random forest and support vector machine (SVM) models, a high classification accuracy can be achieved [12]. However, the accurate extraction of features from respiratory sequences is challenging, especially when dealing with highly noisy signals [13]. Deep learning (DL) models have exhibited superior performance over conventional rule-based algorithms and machine learning models in medical applications, such as arrhythmia detection and diagnosis of retinal diseases from electrocardiogram and retinal images, respectively [14,15]. We previously developed deep learning models to recognize four main types of PVA: double triggering (DT), ineffective inspiratory efforts during expiration (IEE), delayed cycling, and premature cycling under pressure-controlled MV [16,17]. These methods achieved superior classification accuracies compared with conventional methods. However, the model had to be trained with a large amount of labeled data to guarantee its performance, limiting its application to the detection of other types of PVA under different modes of MV. Therefore, developing a high-performance DL model based on a small amount of labeled data remains a challenging task.

Transfer learning, which borrows knowledge from a source domain to facilitate the learning problem in a target domain, provides an effective framework for DL small datasets. In particular, most studies have made use of models pretrained from the large-scale ImageNet database [18], containing 1.2 million images. These models trained from the ImageNet have a strong capability for feature extraction. Thus, they are suitable to be transferred to other fields having small number of image data. Transfer learning has been widely applied to computer vision. However, only a few studies have applied these methods to time series problems in the medical field [19]. These studies require complex time–frequency transformations to convert the one-dimensional (1D) time series to two-dimensional (2D) images, which hampers the application of the algorithm in real time and lacks interpretability [20]. Additionally, such methods have never before been applied to PVA detection. 

To develop a DL model for PVA detection based on a small, labeled dataset, we proposed a transfer learning approach. The approach identified PVA from the 1D respiratory sequences using the 2D–CNN. The 1D breathing cycles were first converted into images. Then, features were extracted from the converted images using pretrained CNN architectures to feed to an SVM for PVA classification. 

## 2. Methods

### 2.1. Overview of the Method

The formal definition of transfer learning involves the concepts of domains and tasks. A domain D consists of a feature space X and an edge probability distribution P(X) denoted by D={X,P(X)}, where {$x_{1},\ldots .,x_{n}$}∈X. The target domain T consists of a label space Y and an objective predictive function f(·), denoted by T={Y,f(·)}, which can be regarded as a conditional probability function P(y|x). It can be learned from the training data, which consist of pairs $\left\{x_{i},y_{i}|x_{i}\in X,y_{i}\in Y\right\}$. Given a source domain $D_{s}$, a learning task $T_{s}$, a target domain $D_{t}$, and a learning task $T_{t}$, transfer learning aims to help improve the learning of the target predictive function f(·) in $D_{t}$ using the knowledge in $D_{s}$. In most cases, the size of $D_{s}$ is significantly larger than that of $D_{t}$. In this work, $D_{s}$ represents the ImageNet dataset and $D_{t}$ represents the respiratory sequence dataset.

**Figure 1:**
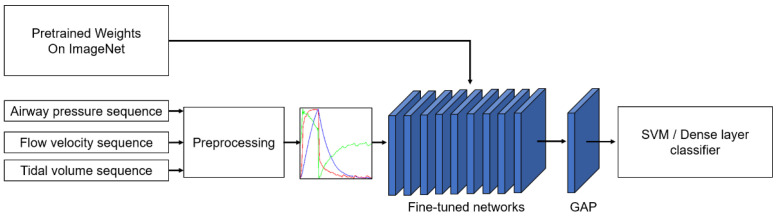
Schematic of PVA identification based on the transfer learning method. The input sequence is handled in the preprocessing section. GAP: global average pooling. SVM: support vector machine.

We proposed a transfer learning approach for PVA classification. Figure 1 shows the schematic of the transfer learning framework. First, the airway pressure, flow velocity, and tidal volume recording were cut into segments, and each data instance was transformed into a red–green–blue (RGB) image via preprocessing. Then, the images were fed into the pretrained deep CNN to extract the features. The features were processed by a global averaging pooling (GAP) layer to generate class activation maps for visual interpretation of the results and fed to an SVM or a dense layer classifier for classification. 

### 2.2. Data Collection and Annotation

Data collection and annotation were performed as described in our previous study [17]. Briefly, adult patients who were admitted to the ICUs of Sir Run Run Shaw Hospital of Zhejiang University and accepted invasive MV were included. Raw respiratory waveform data, including airway pressure sequence and flow velocity sequence, were collected using a ventilator information system (RespCare™, ZhiRuiSi Co. Ltd., Hangzhou, China). The data sampling frequency was 50 Hz. The tidal volume sequence was computed by integrating the flow velocity over time for each breath. All patients were ventilated with PB840 (PB840, Covidien, U.S.) ventilator under assist/control ventilation mode and pressure control type. The study was approved by the ethics committee of Sir Run Run Shaw Hospital of Zhejiang University (No. 20190916-16).

**Figure 2:**
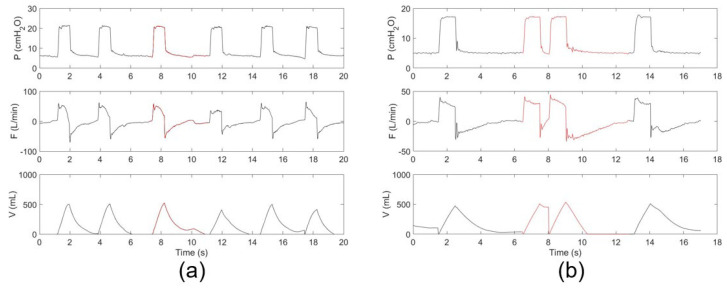
Typical respiratory sequences of (**a**) IEE and (**b**) DT events. The PVA events are indicated in red.

The raw dataset is too large to annotate. We first screened the dataset by selecting ~2% of the recording from each patient to form a dataset with a reasonable size for annotation. The selection ensures that all the subjects contribute to the dataset fairly, according to their MV duration. Details have been described in our previous study [17]. The screened dataset was annotated first by eight junior professionals and finally reviewed by two leading senior professionals. Self-developed software was used for data review and manual labeling. To facilitate annotation, continuous time-dependent respiratory sequences (pressure, flow, and tidal volume) were visualized together with synchronous ventilator settings. Two types of PVA, i.e., DT and IEE, were considered, because they account for most PVA events [21]. DT usually occurs when a patient’s ventilatory center desires a larger breath or a longer inspiratory time than the ventilator setting [22,23]. IEE usually occurs when the patient’s inspiratory effort fails to trigger the ventilator [24]. The information on the screened dataset is summarized in Table 1. The dataset includes three types of sequences, DT, IEE, and OTHER, which include both normal breaths and those showing other types of PVA, condensation, suctions, etc. Figure 2 depicts typical examples of annotated DT and IEE events.

### 2.3. Preprocessing

**Figure 3:**
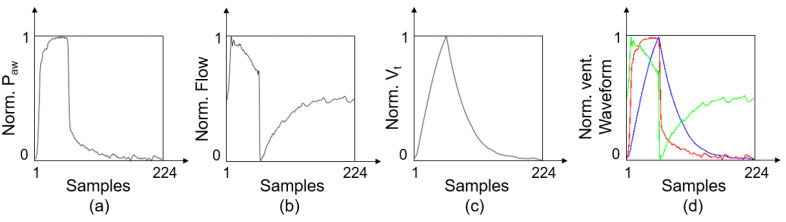
Schematic of the preprocessing. (**a**) Typical P_diag_ represents airway pressure sequence. (**b**) Typical F_diag_ represents flow velocity sequence. (**c**) Typical V_diag_ represents tidal volume sequence. (**d**) Typical RGB image after multichannel fusion.

Because the network used to extract features requires image data as input, we converted the 1D ventilation airway pressure sequence (P), flow velocity sequence (F), and tidal volume sequence (V) into an airway pressure diagram (P_diag_), flow velocity diagram (F_diag_), and tidal volume diagram (V_diag_), accordingly. Preprocessing was performed in the following steps:

(1) Standardization. First, the P, F, and V of each breath were interpolated and resampled to a uniform length of 224. Then, each respiratory sequence was normalized according to Equation (1), where $X_{norm}$ denotes the normalized data, X denotes the original data, and $X_{max}$ and $X_{min}$ are the maximum and minimum values of the original data set, respectively.
  (1) $$X_{norm}=\frac{X-X_{min}}{X_{max}-X_{min}}$$
(2) Dimensional Transformation. Each 1D respiratory sequence was plotted in a 224 × 224 grayscale image, as shown in Figure 3.
(3) Multichannel Fusion. The P_diag_, F_diag_ and V_diag_ were treated as three respective channels and combined into an RGB image.

### 2.4. Pretrained Models for Feature Extraction

The CNN-based transfer learning framework was used in this study as a pretrained model. We adopted three types of CNN models (i.e., MobileNet, VGG 16, and Inception–ResNetV2) for feature extraction, as they represent light-weighted, moderate-weighted, and heavy-weighted feature extractors, respectively. MobileNet is a light-weight model designed to run DL models on mobile devices [25]. It uses depth-wise separable convolution to increase the computing efficiency with only a small reduction in accuracy. VGG 16 is proposed by the Oxford University Visual Geometry Group (VGG) in the context of ILSVRC in 2014. It has been greatly improved for the width and depth of the AlexNet network [26]. In the VGG network, the concept of a convolutional layer is upgraded to the concept of a convolutional group [27]. Inception–ResNetV2 is a hybrid inception module proposed by Szegedy et al. [28]. The architecture significantly improves the recognition performance of ResNetV2 and Inception V4, and the training speed is significantly improved when tested on the ImageNet dataset. 

The fine-tuning retained the feature extractor of the pretrained network including its weights and trained the network together with task-specific classifier. To visualize the networks for better interpretability, we replaced the last three fully connected layers with GAP layers. The GAP outputs the spatial average of the feature map of each unit in the last convolutional layer. Then, it computes a weighted sum of the feature maps of the last convolutional layer to obtain class activation maps [29]. The model was optimized to obtain the best PVA classification performance. This fine-tuning process enables the new network to learn the advanced features of the target domain. In particular, to accelerate the convergence speed of the VGGNet, we added a batch normalization layer [30] between the convolutional layer and the max-pooling layer of the VGG 16 network, similar to the other two pretrained models. 

Table 2 shows the feature extractor of the three pretrained models. We chose the same input size in the experiment for a more reasonable comparison. The feature sizes of MobileNet, VGG 16 and Inception–ResNetV2 are 1024, 1024, and 1536, respectively. 

### 2.5. Performance Evaluation

**Figure 4:**
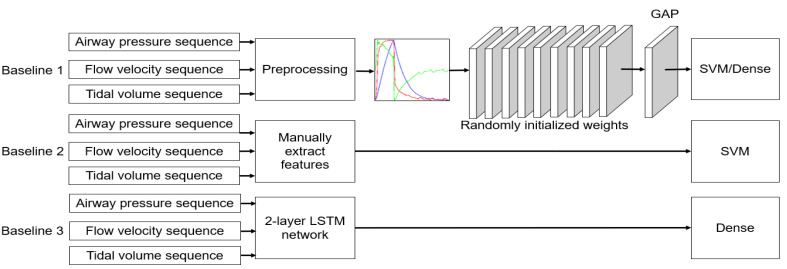
Schematic of PVA identification based on baseline methods.

We used three types of baselines for performance evaluation. The frameworks are shown in Figure 4. First, we compared the evaluation using the CNN networks initialized with random weights rather than pretrained weights. Second, we evaluated whether our method outperformed conventional machine learning methods. The conventional method took 34 time–domain handcrafted features as inputs following the literature [31] and made the classification decision using an SVM with Gaussian kernel. Third, we compared it with our previous study that used a two-layer LSTM network for PVA recognition [17]. The specific feature extractors and classifiers used in this paper are listed in Table 3. 

**Figure 5:**
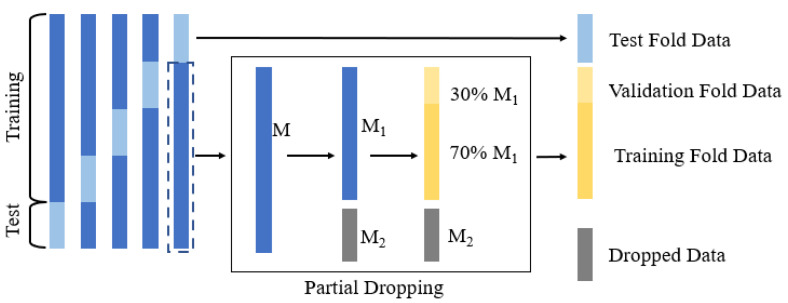
Strategy of partial dropping k-fold cross-validation.

General k-fold cross-validation is a technique of stabilizing the performance of statistical models. The entire dataset was divided into k different subsets, and training was repeated with k − 1 subsets, and evaluation was performed with one subset until all k subsets were evaluated. To examine the influence of the number of training samples on the experimental results, we proposed a partial dropping cross-validation strategy to evaluate the performance of the transfer learning framework. This strategy is illustrated in Figure 5. First, we divided the dataset into k folds and took the data of k − 1 folds to form a subset M. The left fold was used for the testing. Then, part of subset M was dropped ($M_{2}$), and the retained part was denoted as $M_{1}$. There is a partial retention rate (PRR) such that i=[PRR×n ], where [·] means taking an integer. i is the size of $M_{1}$, and n is the size of M. The model was further trained using $M_{1}$, with 70% used for training and 30% used for validation. This procedure was repeated k times for cross-validation. In this study, we set k to 5.

Categorical cross-entropy was adopted as the loss function, and the labels of all data were one-hot encoded. Early stopping and batch normalization were applied to avoid overfitting the model. Batch normalization [30] allows us to use much higher learning rates. Early stopping [32] is an effective strategy of preventing overfitting. During the training phase, 30% of the training data was reserved as a validation dataset. When the validation loss did not decrease for 10 consecutive epochs, the learning rate was automatically reduced. If the validation loss no longer decreased, the training was stopped, and the model was saved as the best one.

The classification accuracy, sensitivity, specificity, and F1 score were calculated to evaluate the performance of the networks:

(2) $$Accuracy=\frac{TP+TN}{TP+TN+FP+FN}$$

(3) $$Sensitivity=\frac{TP}{TP+FN}$$

(4) $$Specificity=\frac{TN}{TN+FP}$$

(5) $$F1=2\times \frac{Sensitivitiy\times Specificity}{Sensitivity+Specificity}$$

where TP, TN, FP, and FN denote the number of true positive, true negative, false positive, and false negative samples, respectively. These classifiers and CNN models were deployed in the Python language with TensorFlow [33]. The network’s fine-tuning was implemented with Keras, with TensorFlow backend.

## 3. Results

**Figure 6:**
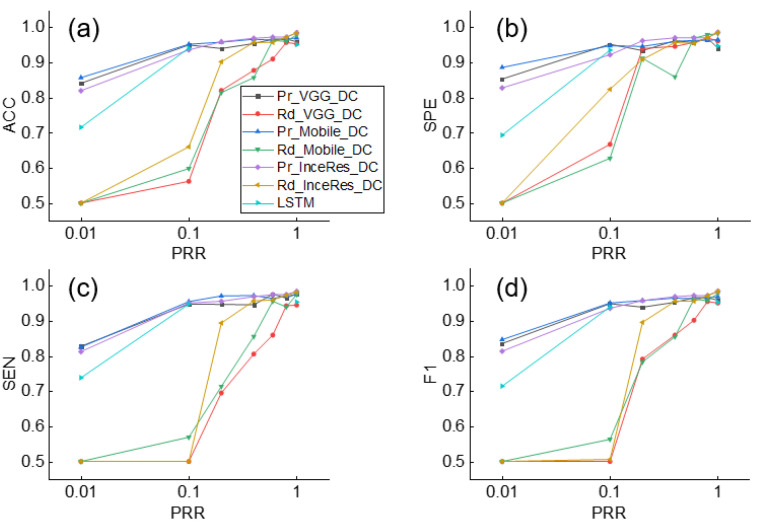
Performance of IEE detection using Pr_CNN_DC, Rd_CNN_DC, and LSTM under different values of PRR. (**a**) Accuracy, (**b**) Sensitivity, (**c**) Specificity, (**d**) F1-score of classification results. ACC: accuracy, SEN: sensitivity, SPE: specificity, F1: F1-score.

Figure 6 shows the results of identifying the IEE for the Pr_CNN_DC and Rd_CNN_DC models. When PRR = 1, the performances using the pretrained models and the randomly initialized models were comparable (F1 score: 0.958, 0.968, and 0.984 for Pr_VGG_DC, Pr_Mobile_DC, and Pr_InceRes_DC, respectively and 0.95, 0.977, and 0.983 for Rd_VGG_DC, Rd_Mobile_DC, and Rd_InceRes_DC, respectively). As shown in Figure 6d, the F1 scores of the Rd_CNN_DC model decreased significantly when PRR dropped, whereas the F1 scores of the Pr_CNN_DC model did not fluctuate. With the decrease in PRR, the gap in accuracy between the Pr_CNN_DC and Rd_CNN_DC models is widened. When PRR dropped to 0.1, the F1 scores of the Pr_VGG_DC, Pr_Mobile_DC, and Pr_InceRes_DC models were 0.948, 0.950, and 0.935, respectively, whereas the F1 scores of Rd_CNN_DC were 0.5, 0.563, and 0.506, respectively. When PRR was further reduced to 0.01, the transfer learning method obtained F1 scores of 0.835, 0.846, and 0.772, respectively, whereas the Rd_CNN_DC model failed to converge. 

**Figure 7:**
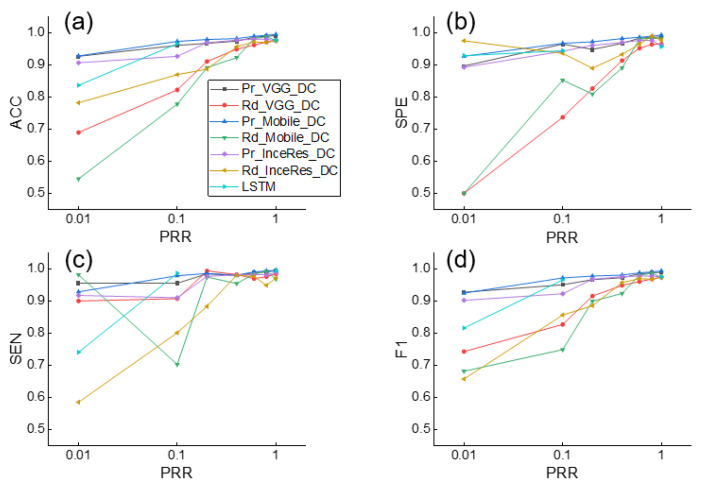
Performance of DT detection using Pr_CNN_DC, Rd_CNN_DC, and LSTM under different values of PRR. (**a**) Accuracy, (**b**) Sensitivity, (**c**) Specificity, (**d**) F1-score of classification results. ACC: accuracy, SEN: sensitivity, SPE: specificity, F1: F1-score.

Simultaneously, the F1 score of the LSTM was only 0.714, which means that the DL method without transfer learning was not as accurate as the transfer learning method on small datasets. Similar findings are shown in Figure 7, which shows the results of the DT identification.

**Figure 8:**
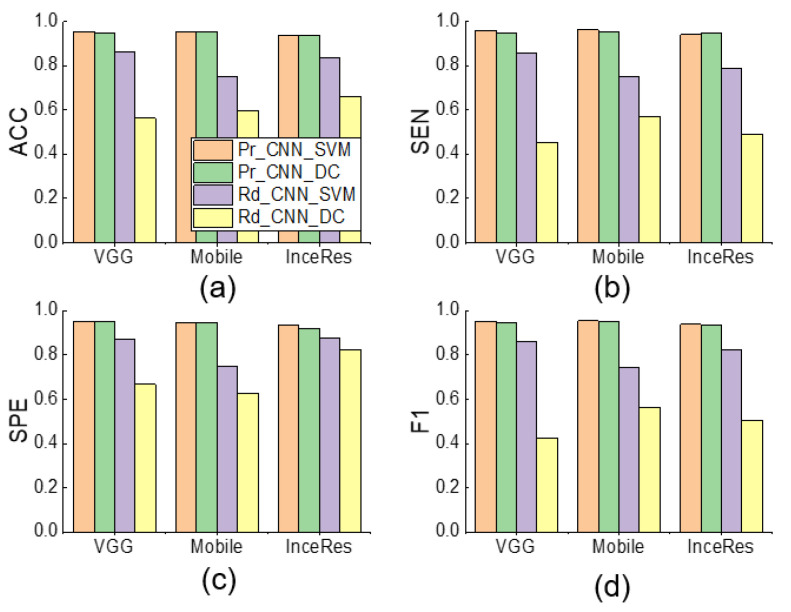
Performance comparison among Pr_CNN_DC, Pr_CNN_SVM, Rd_CNN_SVM, and Rd_CNN_DC of IEE detection using respective features extraction and classification methods (PRR = 0.1). (**a**) Accuracy, (**b**) Sensitivity, (**c**) Specificity, (**d**) F1-score of classification results. ACC: accuracy, SEN: sensitivity, SPE: specificity, F1: F1-score.

**Figure 9:**
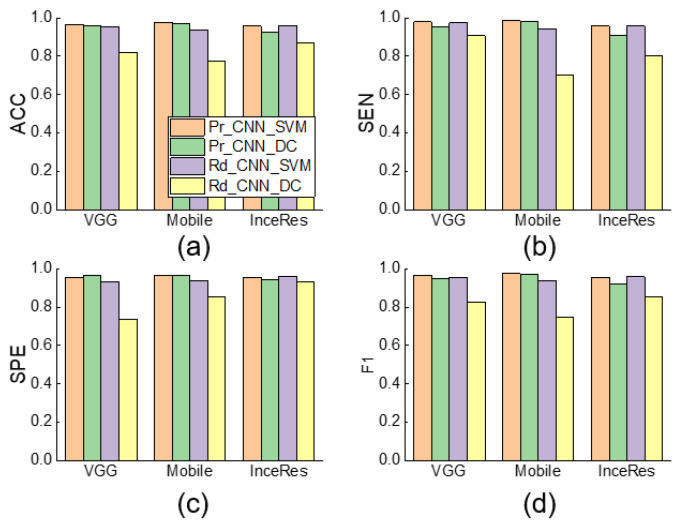
Performance comparison among Pr_CNN_DC, Pr_CNN_SVM, Rd_CNN_SVM, and Rd_CNN_DC and NonTrans-Dense of DT detection using respective features extraction and classification methods (PRR = 0.1). (**a**) Accuracy, (**b**) Sensitivity, (**c**) Specificity, (**d**) F1-score of classification results. ACC: accuracy, SEN: sensitivity, SPE: specificity, F1: F1-score.

When the PRR is set to 0.1, Figure 8 shows the performance comparison among Pr_CNN_DC, Pr_CNN_SVM, Rd_CNN_SVM, and Rd_CNN_DC. It can be seen that the SVM classifier was slightly better than the dense classifier. We found a trivial difference between the results under Pr_CNN_DC and Pr_CNN_SVM, implying that the choice of classifier was not very important in identifying PVA using the transfer learning method. Figure 9 also illustrates the same conclusion. 

Table 4 and Table 5 show the comparison between the models using the SVM classifier. It was observed that the Pr_CNN_SVM model obtained the best performance, whereas the Manual_SVM model gave the lowest F1 score. The results indicate that the CNN-based feature extractor performed better than manual feature selection in the task of PVA detection. 

**Figure 10:**
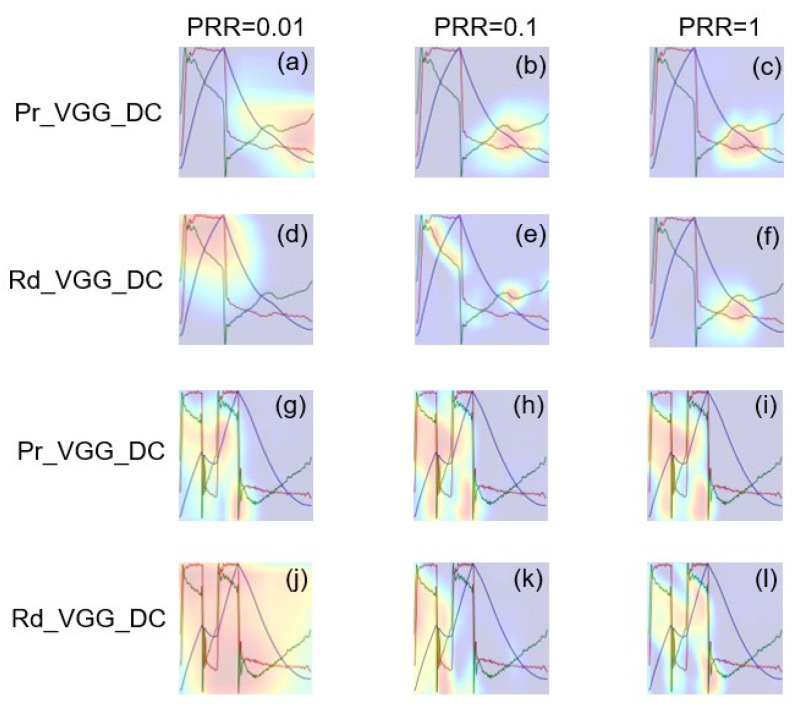
Class activation maps of Pr_VGG_DC and Rd_VGG_DC in identifying IEE and DT waveform. (**a**–**f**) Class activation maps of IEE color image. (**g**–**l**) Class activation maps of DT color image.

Figure 10 shows the class activation maps of Pr_VGG_DC and Rd_VGG_DC. The highlighted part in red represents the region in which the model focused on classification. It was found that, when PRR = 1, the highlighted parts in Pr_VGG_DC and Rd_VGG_DC were both positioned in the key area for identifying the IEE. However, when PRR was reduced to 0.01, the highlighted part was scattered in Rd_VGG_DC but was still concentrated in the key area in Pr_VGG_DC. Similar findings were observed for DT classification.

## 4. Discussion

There is an urgent requirement for the study of ventilatory support techniques during the coronavirus disease 2019 (COVID-19) pandemic, and automatic detection of PVA is one of the challenges faced by clinics worldwide [34]. Using DL approaches to detect PVA is promising, but it is hampered by the limited number of annotated data [26]. We proposed and evaluated a method that applies transfer learning to identify PVA. We transferred three CNN models, which were pretrained based on the large-scale ImageNet database, to extract features from the 1D respiratory sequences. We then adopted different classifiers to recognize two types of PVA, i.e., DT and IEE. Using a partial dropping cross-validation, we found that, with a reduction in the size of the training dataset, the accuracy of PVA classification dropped significantly when using non-transfer learning. In contrast, the transfer learning method achieved a high classification accuracy, even when only 1% of the samples in the original training dataset were involved in training. This indicates the feasibility of transfer learning for identifying PVA cycles having small, annotated datasets.

In developing computerized algorithms for PVA detection, feature extraction and classifier selection are key steps. Sottile et al. proposed using 34 features to classify DT, IEE, flow-limited, premature ventilator-terminated, and synchronous breaths [31]. We adopted their respective extracted features and fed them to the SVM model. The performance was weaker than that of the DL method, probably because the extraction of handcrafted features is vulnerable to noise. We set up three pretrained CNN having different levels of weight to extract the features. The difference in the performance of the three models was less than 5%, which means that even the light-weighted MobileNet could effectively extract the crucial features for PVA analysis. Its strength in feature extraction mainly benefits from the complex image classification problem on the ImageNet dataset. The results also imply that the feature extraction model can be further simplified to achieve satisfactory performance. 

Figure 6 and Figure 7 indicate that the complexity of the model structure was more important than the number of parameters. The structures of extractor in both MobileNet and Inception–ResNetV2 contained more regularization and other methods to enhance the generalization ability of the model. In contrast, although we added the batch normalization layer to the VGG 16 extractor, the generalization ability of the model was weaker than that of the MobileNet and Inception–ResNetV2 extractors. The classifier plays only an auxiliary role in identifying PVA. Figure 8 shows that the models using SVM or dense classifiers obtained similar results. 

The proposed preprocessing method was easy to implement and was reasonable for our research. There are some methods that converted 1D time series to 2D images for CNN processing over the past decade. Some of them transformed the 1D time series into spectrograms using wavelet transforms, Fourier transforms, etc. [19,35], whereas others obtained time–domain characteristics by plotting the waveforms directly onto a canvas [36,37]. The former strategy aimed to highlight the time–frequency characteristics, whereas the latter focused on the original time–domain information. We adopted the latter type of preprocessing, because PVA occurs because of the asynchronous process of human–machine interaction and may not reflect specific frequency properties. However, it has to be pointed out that the resampling procedure is likely to down-sample the waveform signal and may lose the detailed features in the ventilator waveform, particularly for long breathing cycles. Moreover, conversion of the 1D waveform into 2D image may distort the amplitude of the ventilator waveform. Despite of the satisfactory performance of the proposed approach, the influence of the preprocessing method on the results needs to be investigated further. 

The transfer learning shows superior performance over the compared machine learning and standard DL models in PVA detection. By embedding the algorithm into a ventilator information system, which collects real-time ventilator waveforms, the algorithm is promising to detect the occurrence of PVA in real-time under specific ventilation mode to alert the clinicians to deal with it. However, more efforts are required to apply the proposed approach in real clinical settings. First, it remains to be investigated whether the approach can be extrapolated to more ventilator vendors, more ventilation modes, and more PVA types. The method is expected to be applicable to these conditions, because the feature extraction is automatic and thus suitable for detecting other types of PVA under various ventilation modes. Second, we converted the 1D time series into images having a unique size as required by the CNN models. This led to distortion of waveforms at different lengths. Although most breaths last for 4–6 s and 224 samples were sufficient to represent the characteristics of the normal and PVA cycles (longer than previously reported 150 samples for CNN [37]), the influence of the resampling processing should be investigated in the future. Future studies are required to apply this approach in real clinical settings.

## 5. Conclusions

We presented a PVA detection and classification method based on MV sequences. This method exploited CNN-based learning features through transfer learning to fine-tune the pretrained CNN and then classify the features. The results suggest that transferring pretrained 2D–CNN to solve the 1D problem is capable of acquiring a high accuracy in PVA detection with a small dataset. Future application of the transfer learning technology could assist the detection of other types of PVA cycles under various ventilation modes, and therefore, rendering better patient–ventilator interaction, which probably benefits the intubated patients in the ICU, including the COVID-19 patients.

## Figures and Tables

**Table 1:** Respiratory sequences data description.

Pretrained Model	No. of Respiratory Sequences
PVA–DT ^a^	20,398
PVA–IEE	26,453
OTHER ^b^	470,770
Total ^c^	516,210

^a^ Two consecutive breaths with a DT label form a DT event. ^b^ Include normal sequences, other PVA types (flow and cycling asynchronies), and other problems such as condensation, elevated airway resistance, and active exhalation. ^c^ The sum of PVA–DT, PVA–IEE, and OTHER does not equal to Total, because some cycles have two labels. For example, the second breath of a DT event could also be a PVA–IEE.

**Table 2:** Three pretrained networks used in the study.

Pretrained Model	No. of Weights	No. of Features	Input Image Size
MobileNet	3,230,914	1024	224 × 224
VGG-16 network	14,721,602	1024	224 × 224
Inception–ResNetV2	54,339,810	1536	224 × 224

**Table 3:** Feature extractors and corresponding classifiers used in this paper. Pr: use pretrained parameters for the feature extractors. Rd: use randomly initialized weights for the feature extractors. Mobile, VGG, and InceRes represent MobileNet, VGGNet, and Inception–ResNetV2 as the feature extractor, respectively. DC: dense classifier. SVM: SVM classifier.

Type	Model Name	Feature Extractor	Classifiers
Pr_CNN_DC	Pr_Mobile_DC	Pretrained MobileNet extractor	Dense
	Pr_VGG_DC	Pretrained VGGNet extractor	Dense
	Pr_InceRes _DC	Pretrained Inception–ResNetV2 extractor	Dense
Pr_CNN_SVM	Pr_Mobile_SVM	Pretrained MobileNet extractor	SVM
	Pr_VGG_SVM	Pretrained VGGNet extractor	SVM
	Pr_InceRes _SVM	Pretrained Inception–ResNetV2 extractor	SVM
Rd_CNN_DC	Rd_Mobile_DC	MobileNet extractor with r.i.w. *	Dense
	Rd_VGG_DC	VGGNet extractor with r.i.w.	Dense
	Rd_InceRes_DC	Inception–ResNetV2 extractor with r.i.w.	Dense
Rd_CNN_SVM	Rd_Mobile_ SVM	MobileNet extractor with r.i.w.	SVM
	Rd_VGG_ SVM	VGGNet extractor with r.i.w.	SVM
	Rd_InceRes_ SVM	Inception–ResNetV2 extractor with r.i.w.	SVM
	Manual_SVM	Manual feature design	SVM
	LSTM	2-layer LSTM network	Dense

*: r.i.w. randomly initialized weights.

**Table 4:** Results of Pr_CNN_SVM, Rd_CNN_SVM and Manual_SVM model in detection of DT (PRR = 0.1). The result value of Pr_CNN_SVM model is the average of Pr_Mobile_SVM, Pr_VGG_SVM, and Pr_InceRes_SVM. The result value of Rd_CNN_SVM model is the average of Rd_Mobile_SVM, Rd_VGG_SVM, and Rd_InceRes_SVM. ACC: Accuracy, SEN: Sensitivity, SPE: Specificity, F1: F1-score.

	ACC	SPE	SEN	F1
Manual_SVM	0.921 ± 0.004	0.946 ± 0.003	0.895 ± 0.005	0.918 ± 0.004
Pr_CNN_SVM	0.966 ± 0.005	0.958 ± 0.007	0.973 ± 0.009	0.965 ± 0.005
Rd_CNN_SVM	0.949 ± 0.012	0.943 ± 0.009	0.957 ± 0.018	0.949 ± 0.013

**Table 5:** Results of Pr_CNN_SVM, Rd_CNN_SVM, and Manual_SVM model in detection of IEE (PRR = 0.1). The result value of Pr_CNN_SVM model is the average of Pr_Mobile_SVM, Pr_VGG_SVM, and Pr_InceRes_SVM. The result value of Rd_CNN_SVM model is the average of Rd_Mobile_SVM, Rd_VGG_SVM, and Rd_InceRes_SVM. ACC: Accuracy, SEN: Sensitivity, SPE: Specificity, F1: F1-score.

	ACC	SPE	SEN	F1
Manual_SVM	0.638 ± 0.008	0.628 ± 0.019	0.650 ± 0.008	0.637 ± 0.005
Pr_CNN_SVM	0.949 ± 0.004	0.945 ± 0.005	0.954 ± 0.009	0.949 ± 0.005
Rd_CNN_SVM	0.815 ± 0.040	0.830 ± 0.064	0.799 ± 0.037	0.809 ± 0.037

## Data Availability

The data presented in this study are available on reasonable request from the corresponding authors.
